# A Case of Adult-Onset Recurrent Painful Ophthalmoplegic Neuropathy With Bilateral Ophthalmoplegia

**DOI:** 10.7759/cureus.54683

**Published:** 2024-02-22

**Authors:** Hyunji Koo, Katie Tsai, Claire Lee, Ibrahim Mustafa

**Affiliations:** 1 Neurology, Carle Illinois College of Medicine, Urbana, USA; 2 Neurology, Carle BroMenn Medical Center, Normal, USA

**Keywords:** cranial nerve enhancement, hemiplegic migraine, ophthalmoplegic migraine, migraine, recurrent painful ophthalmoplegic neuropathy

## Abstract

Recurrent painful ophthalmoplegic neuropathy (RPON), previously known as ophthalmoplegic migraine, is a disorder typically characterized by recurrent episodes of unilateral headache concurrent with ipsilateral ocular cranial nerve paresis which primarily affects children. Diagnosis is mostly one of exclusion, based on clinical symptoms, supplemented by imaging for enhanced or distorted oculomotor nerves. We present a case of RPON in a 24-year-old adult female with unique features of unilateral left headache with ipsilateral pupillary dilation spreading to bilateral dilation and no MRI findings of oculomotor nerve enhancement.

## Introduction

Recurrent painful ophthalmoplegic neuropathy (RPON), formerly known as ophthalmoplegic migraine, is a rare disorder typically characterized by recurrent episodes of unilateral headache associated with ipsilateral ophthalmoplegia due to involvement of the third, fourth, or sixth cranial nerves [1]. Formerly classified as a migraine, it is now classified by the International Classification of Headache Disorders (ICHD) as a type of neuropathy and facial pain [1]. Although RPON is most commonly seen in children, adult cases have also been documented [2,3]. The etiology of RPON has no clinical consensus, but it is speculated to be related to nerve inflammation, nerve demyelination, or neurovascular compression [4-6]. Treatment with corticosteroids has been shown to be beneficial in some patients [1]. Other potentially successful treatment options have also included first-line migraine prophylactic agents such as valproate and topiramate [4]. Here, we present a case of RPON in a 24-year-old female who was treated with a high-dose prednisone taper, leading to a gradual improvement of her symptoms.

## Case presentation

A 24-year-old female with a history of hemiplegic migraines presented to the hospital with intractable left-sided headache, blurry vision in both eyes, and left-sided weakness typically associated with her hemiplegic migraine. She noticed her pupils were dilated, initially only on the left side, then became bilateral over the next few hours. She also reported pain with eye movements, nausea, and photophobia. She denied fever, numbness, or tingling in her extremities. She tried ubrogepant 200 mg with no significant relief. She had a history of being hospitalized several times in the past two years for similar complaints, most recently a month prior.

Her chart showed extensive workup, including eight brain CTs over the past two years, numerous Brain and Neck computed tomography angiographies (CTAs), brain MRIs, lumbar punctures, EEG, and MRI orbits, all of which were negative. Evaluation by neuro-ophthalmology was also unremarkable with no concern for optic neuritis or optic neuropathy. In the past, she had been given prochlorperazine, diphenhydramine, ketorolac, dexamethasone, acetaminophen, sumatriptan, and magnesium with varied results. She also tried pilocarpine drops to treat pupillary dilation with no improvement. On her last admission, her symptoms gradually improved with methylprednisolone 250 mg daily, prochlorperazine 10 mg three times daily, and diphenhydramine 25 mg three times daily. She was discharged with topiramate 25 mg daily and ubrogepant 100 mg as needed as an abortive therapy.

Her past medical history was significant for hemiplegic migraines with associated anisocoria, anxiety, chronic marijuana use, vitamin D deficiency, and vitamin B12 deficiency. Her medications at the time included topiramate 25 mg twice daily, ubrogepant 100 mg as needed, trazodone 50 mg daily, venlafaxine 37.5 mg daily, cholecalciferol 25 mcg daily, and B12 2,000 mcg daily. She denied cigarette or alcohol use but did report chronic marijuana use. There was a family history of migraine in her mother, but there was no other family history of similar eye disturbances or neuropathy.

**Figure 1:**
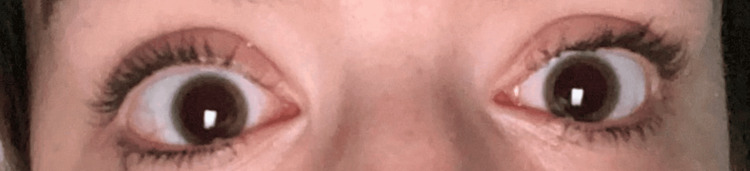
Patient presented with pupils that were fixed dilated 8 mm bilaterally.

**Figure 2:**
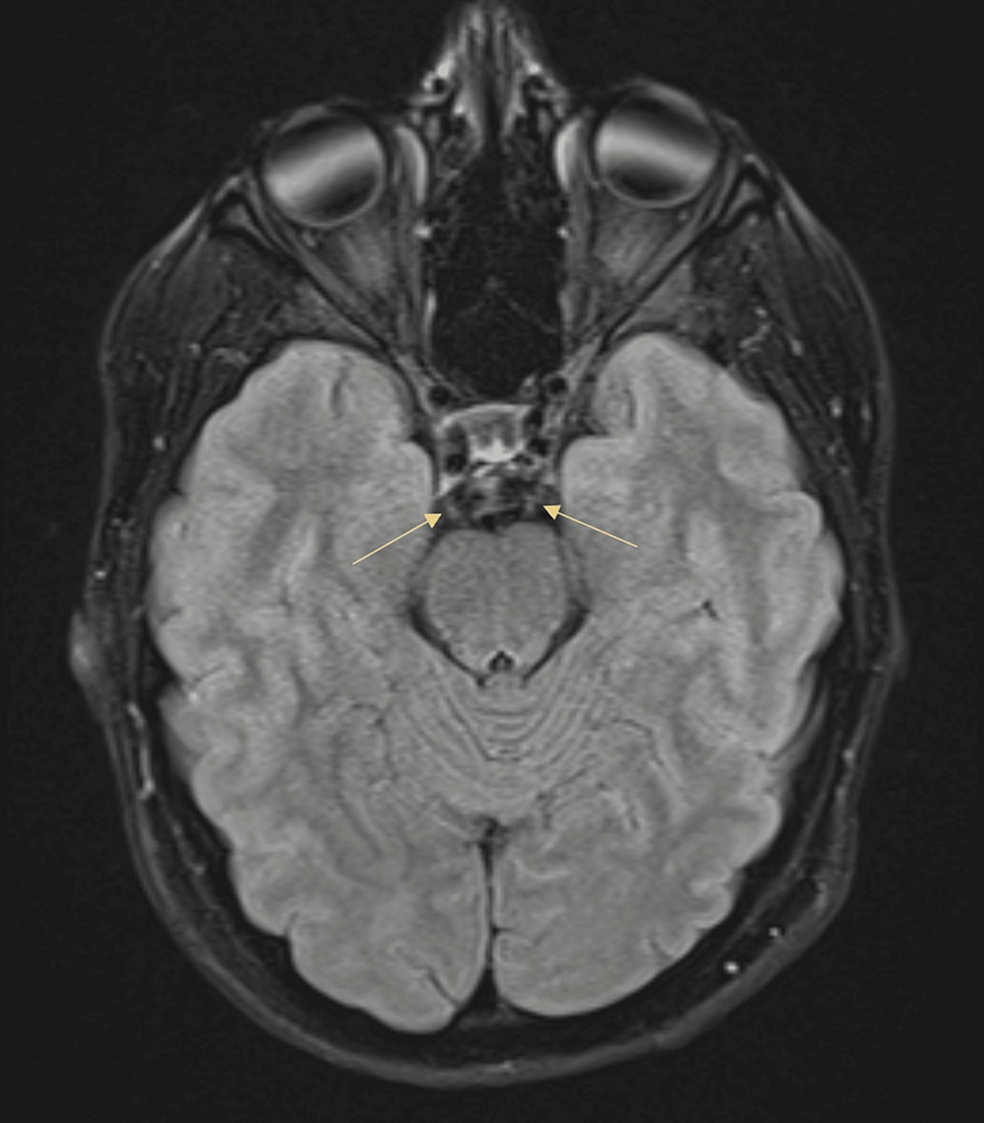
FLAIR axial image of brain MRI showing no enhancement of oculomotor nerves (yellow arrows).

On exam, vital signs were normal. Her pupils were fixed and dilated 8 mm bilaterally (Figure 1). Strength in her upper and lower extremities was 4/5 on the left and 5/5 on the right. The rest of the physical and neurological exam was unremarkable. Labs were all within normal range, including complete blood count (CBC) and comprehensive metabolic panel (CMP). Brain CT and MRI were negative for acute findings. MRI of the internal auditory canal (MRI-IAC) was also negative (Figure 2).

Based on her presentation, she was diagnosed with an unusual case of RPON. She was started on methylprednisolone 250 mg four times daily for three days, which led to significant improvement in her symptoms. On the day of discharge, she was feeling close to her baseline and was transitioned to oral prednisone 60 mg daily with a subsequent 10 mg taper each week with outpatient follow-up.

## Discussion

Once classified as a migraine, RPON is now considered a type of neuropathy [1]. This corroborates our patient’s observed improvement of symptoms through a steroid regimen for six weeks. While she was on a high dose of methylprednisolone in the hospital and then on a prednisone taper after discharge, she had no recurrence of her migraines for eight months. 

The etiology of RPON has no consensus. Some propose nerve demyelination [4,5] or neurovascular compression [6] as potential mechanisms, possibly due to an underlying genetic disorder, ischemic disease, trauma, viral infection, or inflammatory disorder [7]. This can sometimes be detected through nerve enhancement of the oculomotor nerve on MRI. A pooled data analysis of 165 RPON patients showed that this feature appeared in 27.6% of patients with RPON who received enhanced MRIs [4]. In adult-onset patients, 34.6% of patients had enhanced MRIs that demonstrated abnormalities in at least one cranial nerve with cranial RPON [4]. In our patient, MRI-IAC found no nerve thickening or enhancement of the oculomotor nerves. However, some have noticed that imaging abnormalities could not be seen during interictal periods or during a first-time MRI, and could only be detected on MRI after numerous recurrent episodes [8]. Thus, negative studies do not rule out RPON.

Our patient also presented with bilateral mydriasis, an unusual symptom of RPON. ICHD classification defines criteria for RPON as (1) at least two attacks of a unilateral headache with ipsilateral paresis of one or more ocular motor nerves, (2) with orbital, parasellar, or posterior fossa lesions excluded, and (3) no other ICHD-3 diagnosis that better accounts for symptoms [1]. Liu et al propose modifying this diagnostic criteria to (1) at least two unilateral headache attacks ipsilateral to ophthalmoplegia developed at approximately the same time or at least 15 days before, and (2) clinical signs of paresis of one or more cranial nerves or enhancement of motor nerves on MRI, and (3) no other ICHD-3 diagnosis that better accounts for symptoms [4]. Our patient met both sets of these criteria, with numerous recurrent episodes of a unilateral headache on her left side with concurrent ipsilateral eye dilation indicating ophthalmoplegia. During some of her attacks, she would experience unilateral headaches with ipsilateral dilation spreading to bilateral dilation. Thus, this review suggests considering RPON for diagnosis for patients with at least one migraine concurrent with bilateral dilation, not just unilateral.

Adult-onset cases of RPON are also not as common but have been reported in the literature. Liu et al found the mean age of onset of RPON to be 22.1 years of age, with 34.2% of patients with an onset age of over 18 [4]. A 40-year-old male with a severe unilateral migraine headache of long duration was diagnosed with RPON and, three days later, developed ipsilateral ophthalmoplegia, both of which subsided with a tapering dose of oral steroids and migraine prophylaxis [2]. Diplopia, pain with eye movement, and nodular enhancement seen on MRI orbit of a 50-year-old female with RPON were resolved with 20 mg of oral prednisone twice daily for six months [3]. A 48-year-old woman who had ocular nerve palsy and migraine attacks every 1-2 months was diagnosed with RPON, and like our patient, her symptoms resolved immediately with prednisolone [9]. 

Optimizing treatment for RPON is essential since symptoms can be debilitating and lifelong. In Liu et al, 76 patients received corticosteroids, of which 96.2% showed rapid improvement compared to those without corticosteroids [4]. However, Li et al recruited eight patients to study different treatment options for RPON with varying results [7]. Three out of four patients receiving glucocorticoid and neurotropic drugs had their symptoms disappear completely within a month. At the same time, two out of three patients who were only observed noted symptom regression within a month, despite any medications. This study suggests symptoms tend to regress after 3-28 days regardless of treatment intervention. The difference in results between these two studies reflects the need for individually tailored treatment regimens for RPON.

It is important to note potential prophylactic therapies for RPON, given that 30% of patients may experience permanent neurological sequelae with repeated attacks such as residual weakness of the cranial nerve and pupillary dysfunction [4]. Antimigraine medications have been commonly prescribed. Vieira et al suggest flunarizine decreased the number of episodes in a 7-year-old patient [10]. A 33-year-old female in Liu et al took amitriptyline 25 mg and sodium valproate 500 mg with no further episodes at the one-year follow-up. Compared to patients taking only antimigraine therapy, those taking a combination of antimigraine medication and corticosteroids had more rapid improvement in symptoms [4]. As this medication regimen is similar to that in our patient, this suggests a promising outlook for the role of antimigraine and corticosteroid medications for the management of RPON. Perhaps a higher dosage or longer duration of steroids could lead to faster or more prolonged recovery. Additional studies are necessary to investigate the etiology and best recommendations for the prevention of RPON episodes.

## Conclusions

Here we present a case of RPON in a 24-year-old female with unilateral left headache with ipsilateral eye dilation spreading to bilateral dilation and no MRI findings of oculomotor nerve enhancement. She responded well to a high-dose prednisone taper, leading to a gradual improvement of her symptoms. Additional studies are necessary to investigate the etiology and best recommendations for the prevention of RPON episodes.
